# Novel homozygous nonsense mutation of *MLIP* and compensatory alternative splicing

**DOI:** 10.1038/s41525-022-00307-y

**Published:** 2022-06-07

**Authors:** Jean Mezreani, Sébastien Audet, Florence Martin, Jade Charbonneau, Valérie Triassi, Eric Bareke, Annie Laplante, Jason Karamchandani, Rami Massie, Colin H. Chalk, Erin O’Ferrall, Martine Tétreault

**Affiliations:** 1 CHUM Research Center, Montreal, Montreal, QC Canada; 2 Department of Neurosciences, University of Montreal, Montreal, QC Canada; 3 Department of Bioinformatics, University of Montreal, Montreal, QC Canada; 4 Department of Neurology and Neurosurgery, Montreal Neurological Institute, Montreal, QC Canada; 5 Department of Pathology, Montreal Neurological Institute, Montreal, QC Canada

**Keywords:** RNA sequencing, Transcriptomics, RNA splicing, Mutation

## Abstract

Despite the growing accessibility of clinical sequencing, functional interpretation of variants remains a major hurdle to molecular diagnostics of Mendelian diseases. We aimed to describe a new adult-onset myopathy with muscle weakness and hyperCKemia caused by a nonsense variant in muscular *LMNA*-interacting protein (*MLIP)*. Following RNA-sequencing, differential expression analysis uncovered a significant downregulation of this gene, which had a surprisingly mild effect on *MLIP* protein expression. RT-PCR and long-read sequencing (LRS) both support an important transcriptome shift in the patient, where decreased *MLIP* levels are seemingly due to nonsense-mediated decay of transcripts containing the exon 5 mutation. Moreover, a compensatory mechanism upregulates the functionally lacking isoforms and generates novel transcripts. These results support the recently discovered clinical implications of *MLIP* variants in myopathies, highlighting for the first time its relevance in adult-onset cases. These results also underline the power of LRS as a tool for the functional assessment of variants of unknown significance (VUS), as well as the definition of accurate isoform profile annotations in a tissue-specific manner.

## Introduction

Myopathies and muscular dystrophies are a large group of progressive genetic neuromuscular diseases that emerge from dysfunctions and irregularities within the muscular tissue, affecting its general integrity, structure, and molecular activity^1^. Gradual degeneration of the muscle fibers prompts variable clinical presentation, generally including but not restricted to; muscular weakness, cramps, stiffness, posture instability, and ultimately leading to a probable loss of motor functions^1^. Due to the notable heterogeneity of these pathologies, the association of putative disease variants with phenotypes is highly complex. This hinders the ability to achieve an official diagnosis, as further genetic and molecular characterization is often required but strenuous. This drawback is amplified in genes that have yet to be extensively studied from a functional standpoint and have not yet been associated with muscle-related disorders.

Muscular *LMNA*-interacting protein (*MLIP*) has only recently been identified as a cause of autosomal recessive myopathy with rhabdomyolysis, myalgia, and elevated serum creatine kinase^2^. The report highlights eight different variants in the *MLIP* gene within seven pediatric biallelic carriers, all of which are expected to cause major protein alterations: four frameshifts leading to premature stop codons, two stop-gains, as well as two alternative splicings which, respectively, induce an exon skipping and a premature stop-codon^2^. While literature concerning the *MLIP* function is not extensive, it is generally thought to play a role in the regulation of transcriptional activators in pathways such as the *Akt/mTOR/FOXO1* cardiac stress response^3,4^ and is known to interact with lamin A/C (*LMNA*)^5^, which is a largely studied cause of muscle diseases^6,7^. Interestingly, all the investigated mutations localize near exon 4 or 9, resulting in a loss of the nuclear localization signal (NLS) and/or the AT-Hook DNA binding motif, respectively^5,8^. It has been suggested that such major changes theoretically result in a considerable functional effect, therefore likely leading to dysregulations of various important biological pathways^2^.

Considering the group had access to a small cohort of patients with similar variants, they were able to demonstrate functional changes through the consistent reduction in mRNA levels of *MLIP*. The quantitative droplet digital PCR suggests nonsense-mediated decay, which should ultimately result in lowered protein levels and guided our initial hypothesis. In a single patient (Z46) exhibiting comparable but rather different phenotypes, a more in-depth functional assessment is required to assess the relevance of *MLIP* as the best candidate to explain the distal myopathy. Hence, on top of standard validation through western blot, quantitative PCR (qPCR), and reverse transcriptase PCR (RT-PCR), we propose long-read sequencing (LRS) as a potent clinical tool to identify accurate tissue-relevant transcripts, as well as being indicative of mRNA level changes in an isoform-specific manner.

## Results

Our patient presented around age 50 with distal (mainly dorsiflexors) more than proximal leg weakness and distal hand weakness. Despite a late-onset of debilitating symptoms, careful history revealed that he had consulted around age 5 for poor sports performance (unable to run as fast as his peers and to ice skate) but never received a clear diagnosis. The absence of muscle disorders or consanguinity in the familial history was also noted. By age 60 he had developed exaggerated lumbar lordosis and a Trendelenburg gait. Muscle pain and cramping were observed but no rhabdomyolysis was reported. Evaluation of basal serum creatine kinase did reveal a significant increase (888 U/L) that lands in the observed hyperCKemia range of 300–3000 U/L^2^. Similar to the pediatric cases, our patient does not exhibit cardiac abnormalities, which could have been expected considering *MLIP* known role in adaptation to cardiac stressors^3^. Muscle biopsy showed only a single necrotic fiber, but many fibers with internal nuclei, fiber-size variability with hypertrophied fibers, type 1 fiber atrophy, and type 2 fibers containing small rimmed vacuoles. EMG demonstrated small polyphasic motor units with some fibrillations and positive sharp waves. ECG, ECHO, and a 24 h Holter monitor were also conducted but the results were unremarkable.

**Fig. 1:**
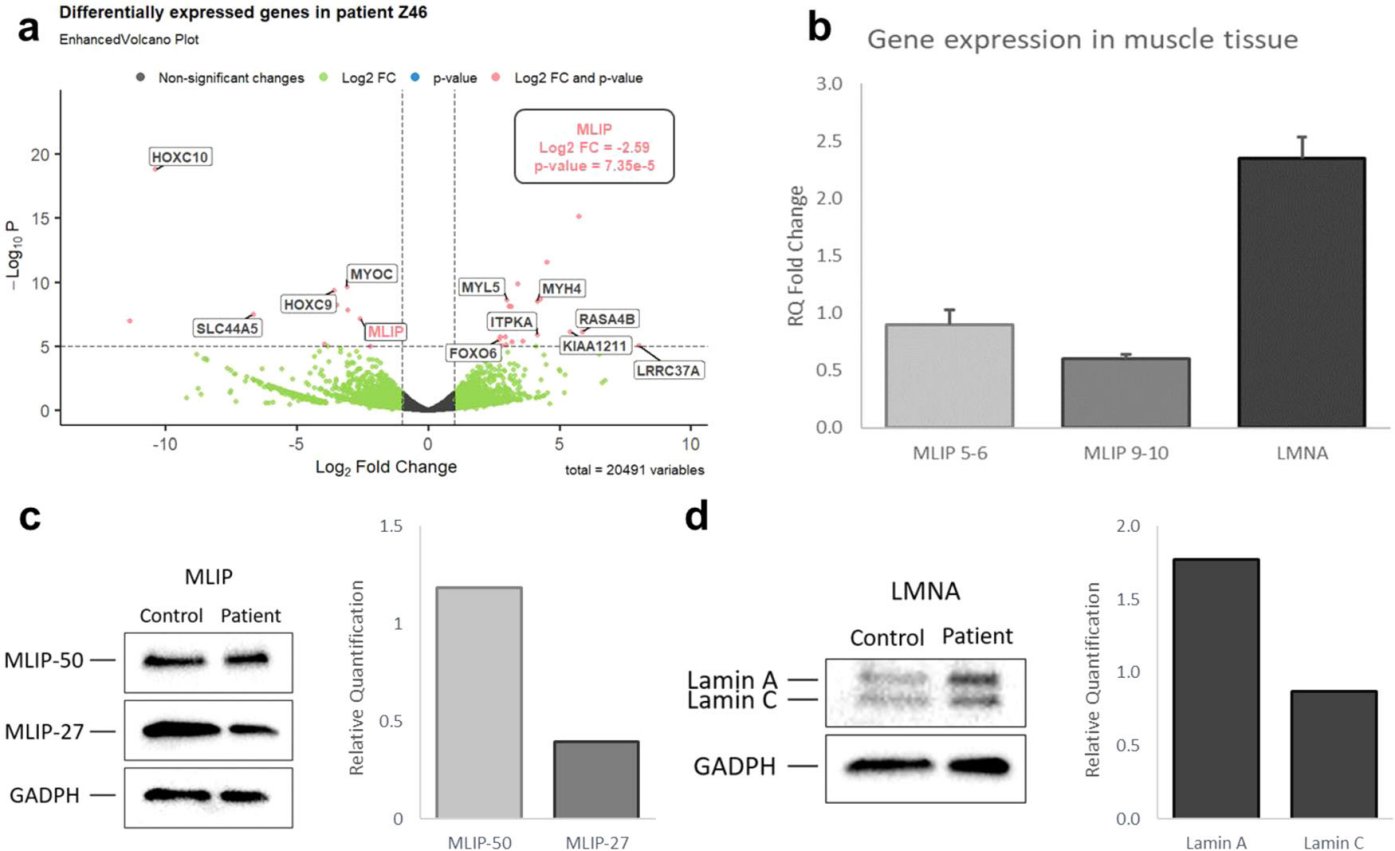
MLIP and LMNA respective expression in muscular tissue. **a** Volcano Plot highlighting the most differentially expressed genes in Z46 compared to controls following DESeq analysis. **b** RT-qPCR reveals mRNA expression levels for the targeted genes: *MLIP* 5–6 accounts specifically for transcripts containing the nonsense mutation, *MLIP* 9–10 covers all the known transcripts, and *LMNA* probe accounts for both lamin A and C. *RPS29* is used for data normalization, error bars correspond to standard deviation (triplicata). **c, d** Western Blot analysis reveals the presence of four isoforms of MLIP, two of which can be quantified (27 kDa and 50 kDa). Both lamin types also appear for LMNA and were analyzed separately. GAPDH is used as the reference gene for normalization of the blot in both instances.

Following inconclusive gene panel testing (Supplementary Table 1)^9^, RNA-sequencing served as a diagnostic tool for this complex case, where variant calling data revealed a nonsense homozygous mutation in the exon 5 of *MLIP* (NM_001281747.2: p.Gln762Ter, c.2284 C > T). This finding was confirmed by Sanger sequencing of the patient’s DNA. Upon performing a differential expression analysis using DESeq, a highly significant decrease can be observed in *MLIP* expression (Fig. 1a)^10^. An exon usage analysis was performed not only to identify alternative splicing in Z46 but also to confirm the global decrease of *MLIP* expression (Supplementary Fig. 2). A pathway enrichment analysis, aimed at identifying additional validation targets and molecular changes, yielded no significant results regarding *MLIP* known functions or main interaction partners^11,12^.

The downregulation of *MLIP* was consolidated using two qPCR probes, targeting either transcript that specifically contains the nonsense variant, or accounting for all isoforms. The latter revealed a similar tendency to the RNA-sequencing: although more modest, a decrease of about 40% of *MLIP* transcripts is observed (Fig. 1b). Interestingly, a considerable upregulation of *LMNA* was also noted. Regarding the western blot, preliminary results showed a correlating downregulation of MLIP for the 27 kDa isoform (ENST00000370876.6), which contains neither exon 4 or 5. A negligible change for the 50 kDa (ENST00000274897.9), which theoretically carries the exon 5 mutation and should not be produced, was unexpected (Fig. 1c). Unfortunately, NLS containing isoforms (ENST00000502396.5 and ENST00000514921.5) could not be quantified, as the extraction was not specific to nuclear proteins, leading to very light bands for both samples (Supplementary Fig. 3). Again, increased levels of LMNA can be observed, especially lamin A, exhibiting a nearly two-fold increase in comparison to control (Fig. 1d).

**Fig. 2:**
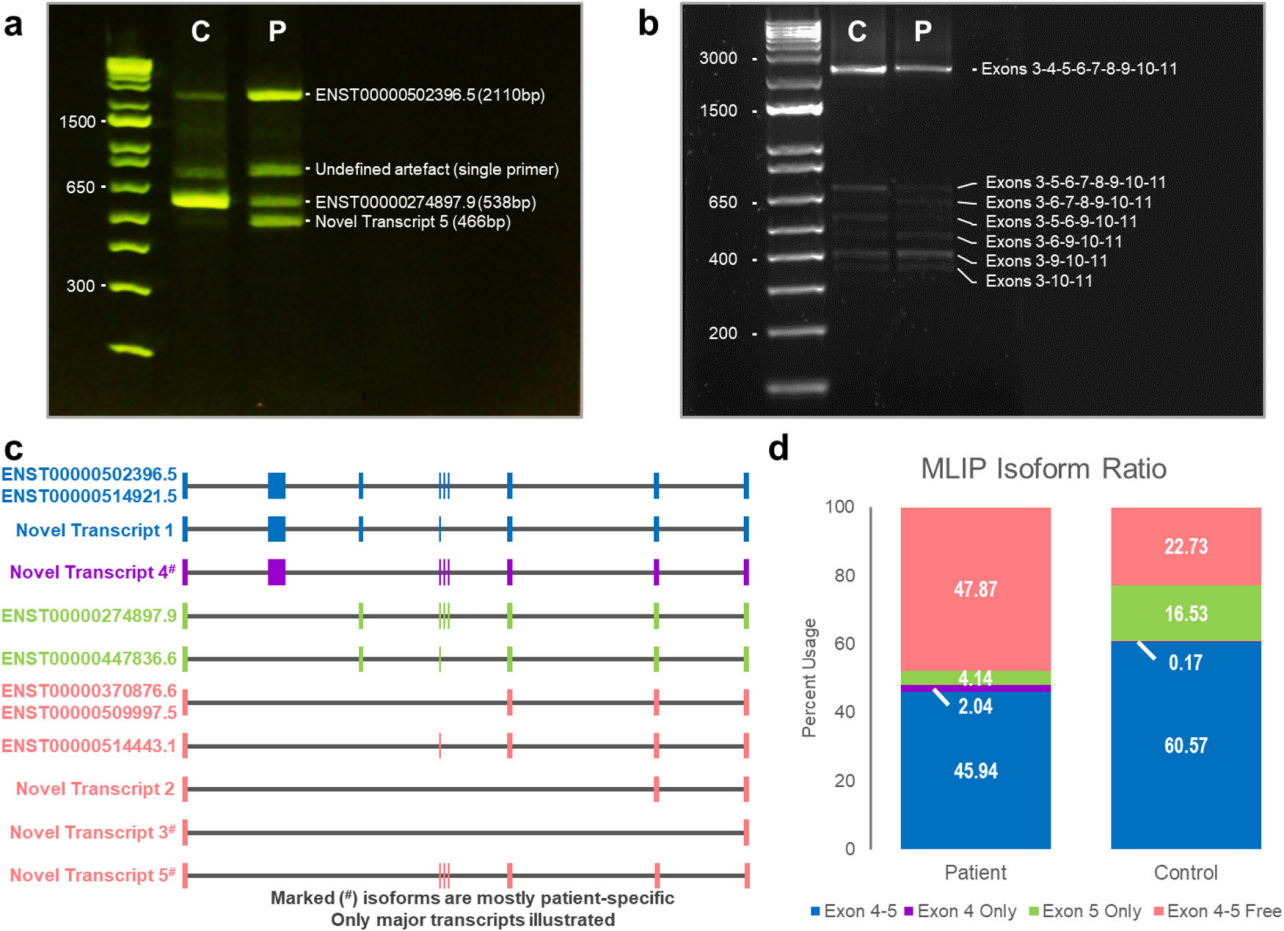
Evaluation of nonsense variant effect on transcript balance of MLIP. **a** Migration of RT-PCR products on 2% E-gel following amplification between exons 3 and 8. 1 Kb Plus E-gel DNA ladder was used for determination of fragment lengths. P = patient, C = Control. **b** Qualitative visualization of 100 ng of sequencing library on a 1% agarose gel with GelRed revealing agent. Migration was done prior to the end prep step, and 1 Kb Plus DNA Ladder served for amplicons length evaluation. **c** Visual representation of major (high confidence) muscle transcripts identified with LRS data. Corresponding annotated transcripts are identified (exons 3-11), and all isoforms are color-coded depending on grouping features. **d** Representation of the samples’ relative transcripts balance, grouped by relevant features. Isoform quantification was performed by FLAIR, and final data contains 82.9k (patient) and 52.3k (control) reads.

RT-PCR was performed between the 3rd and 8th exon of *MLIP*, capturing most muscle transcripts while keeping the interpretation simple. Although qualitative, gel migration revealed a clear alteration of the patient’s transcript balance: while the second to the lowest band is the main isoform in the control, it is considerably decreased in Z46 compared to other visible transcripts, which appear upregulated (Fig. 2a). When correlating amplicons with the GTEx muscle isoforms repertoire, we supposed the presence of transcripts ENST00000502396.5, ENST00000274897.9, and a novel isoform containing exons 3–6–7–8, making it the only fragment that does not carry the variant.

LRS subsequently allowed isoform-specific quantification of all known transcripts. Data reveal a near-perfect correlation with RT-PCR results (Fig. 2b), where a drastic downregulation of isoforms containing exon 5 is milder when exon 4 is co-expressed, and the proportional expression of transcripts that do not carry the mutation is significantly increased (Fig. 2c, d). Additionally, we discovered that the patient seems to produce and conserve novel transcripts that contain exon 4, but not 5. Furthermore, they’re not the only novel transcripts that our analysis revealed: while ENST00000502396.5 and ENST00000370876.6 are indeed the main muscle isoforms, ENST00000447846.6, ENST00000514433.1, as well as two other newly identified transcripts (labeled Novel Transcript 1 and 2) exhibit levels comparable to ENST00000274897.9 in both samples (Fig. 2c). Ratio quantification utilized data from all the isoforms, but only high-confidence transcripts are presented as potential novel isoforms, none of which exhibit alternative splicing that differs from canonical reading frames.

## Discussion

In this report, we used RNA-sequencing in a clinical setting after standard gene panels failed to identify candidates for the patient’s pathology. While variant calling did not find pathogenic mutations in relevant myopathy-related genes (Supplementary Table 1), a novel homozygous variant causing an early truncation of *MLIP* on both alleles was predicted to have a highly negative effect on its function. Although there were very few studies about *MLIP* function, and no links to any pathology, numerous factors hinted at the relevance of this locus to myopathies. A muscle-specific expression pattern and a proven interaction with *LMNA*^5^, combined with the high in silico pathogenicity score as well as the decreased mRNA expression in transcriptomic data prompted further investigation of the variant. Very recently, an article identified *MLIP* as disease-causing in a cohort of patients exhibiting early-onset myopathy with rhabdomyolysis^2^. Similar to the pediatric variants, the premature translation termination in exon 5 is predicted to importantly modify both the structure and the functions of the protein by inducing a biallelic absence of its AT-Hook DNA binding motif^8^. Indeed, current literature states that in addition to being present in two of the three main muscle isoforms, exon 5 is mutually inclusive to transcripts containing exon 4. Considering the latter contains the NLS^5^, the mutation reported in our patient could potentially hinder *MLIP* nuclear role. Nevertheless, it seems the localization of variants affects phenotypic presentation, as clinical examination findings are considerably different between our patient and the younger cohort.

One of the advantages of RNA-sequencing is the functional insight that can be gathered from expression data^13^. It has been hypothesized that dysfunctional *MLIP* potentially disrupts multiple molecular pathways, such as causing an overactivation of the *Akt*/*mTOR* or *FOXO1* pathway in correlation to a reduced adaptive response to stressors^3,4^. Unfortunately, there was no significant enrichment of the genes related to those networks in our data. Similarly, nothing noteworthy can be found in regard to both glycolysis and gluconeogenesis^2^. Observable molecular changes include the activation of the host immune system, which indicates probable progressive necrosis of unhealthy muscle cells^14^. The Syndecan-1 pathway also appears significantly upregulated, which might play a key role in protecting against cardiac phenotypes^15^. While our study gives interesting insights into potential molecular consequences of *MLIP* mutations, the analysis was performed on a single patient, and conclusive results surrounding pathway enrichments require more samples.

As the quantification of mRNA expression is an important step of the functional validation, we made sure to capture changes accurately by using two probes simultaneously. While a larger decrease could have been expected following the differential expression analysis, the results correlate with the general decrease of *MLIP* mRNA in muscle tissues. These non-significative observations support our hypothesis that while *MLIP* mRNA likely undergoes nonsense-mediated decay, the cells attempt to compensate for truncated proteins by specifically splicing transcripts to contain exon 4 and produce the nuclear protein. This would rationalize the considerable upregulation of *LMNA*, presumably as a compensatory mechanism for the deficiency of its direct partner^5^. Regarding protein levels, only two of the four visible isoforms can be quantified but results once again correlate with previous experiments: MLIP expression is decreased, causing upregulation of LMNA to compensate for the likely diminished yet necessary interaction between the nuclear proteins. The presence of unaltered MLIP proteins in the patient was unexpected. While the C-terminal antibody does not allow detection of truncated proteins, normal bands in Z46 prove that muscle cells find a way to produce nearly full-length proteins despite the stop-gain. Two hypothetical explanations for this phenomenon are an elevated proportion of stop-codon read-through in response to reduced functional *MLIP* levels^16^, and alternative splicing allowing non-canonical excision of the exon 5 specifically^17,18^. These partial escape mechanisms, if observed, are likely to play a role in the milder phenotypes of our patient compared to pediatric cases.

It has been shown that distinct isoforms can have tissue-specific functions, hence the aim of evaluating changes in transcripts ratio^19^. The qualitative RT-PCR was key in constructing the hypothesis of conflicting cellular mechanisms: nonsense-mediated decay appears to oppose the upregulation of the functionally relevant *MLIP* transcripts in response to the deficiency of proteins capable of translocating to the nucleus. This came from visible downregulation of transcripts containing exon 5, while other isoforms are promoted in comparison. As transcripts containing the NLS theoretically always carry the variant, they require a substantial transcription upregulation to overcome concurrent mRNA decay.

LRS data confirmed our suppositions, clearly highlighting a significant decrease of transcripts containing the variant as well as a proportional increase of other transcripts, particularly those containing exon 4. In fact, not only is it observable in canonical isoforms, but Z46 also expresses novel transcripts containing exon 4 without its usual splice partner. While those new isoforms represent a minority of the *MLIP* transcriptome, they confirm the hypothesis that compensatory alternative splicing allows the production of nearly full-length proteins. Another unexpected result was that additional unreported non-canonical transcripts were present in non-negligible amounts for both samples. This suggests that their expression would not be restricted to our study and that the tissue-specific alternative splicing of *MLIP* might be even more extensive than what current literature suggests. While further assessment is required to confirm these findings, they prompt the relevance of performing LRS on more samples. Furthermore, we believe the approach is very powerful and could uncover similar results in a broader range of pathologies, underlining its vast potential in clinical contexts. Indeed, while LRS currently offers less accurate base calling than its short-read homolog, its true strength lies in the assembly of *de novo* tissue-specific transcript annotations, which hints toward a considerable improvement of current knowledge regarding existing transcriptomic splicing^20^. While long-read methods are still in an early developmental stage, community enthusiasm has led to consistent improvement surrounding the approach and related bioinformatics tools. Therefore, it would not be surprising if LRS applications reach a largescale, whole-transcriptome level in the not-so-distant future.

Overall, our report of a biallelic nonsense mutation supports the implication of *MLIP* as a causative gene for myopathies with muscle weakness and hyperCKemia. The important element we highlight is that clinicians should consider *MLIP* mutations outside of pediatric cases, as adult-onset phenotypes, albeit milder, could ensue from functional changes in the protein. Indeed, clinical heterogeneity is to be expected in *MLIP*-related disorders, mainly due to its complex transcriptomic architecture. In that regard, LRS shall help define more accurate tissue-specific transcript annotations. Along with future cellular modeling of the mutations, it should give additional insights into the protein molecular functions. We strongly believe our findings should further assist clinicians in the diagnosis of patients carrying *MLIP* variants, but also in the assessment of functional effects of candidate variants, where available transcript annotations are potentially incomplete or inaccurate.

## Methods

### Patient

The proband was evaluated by an experienced neurologist (E.O.) and had a muscle biopsy as part of his clinical workup. The McGill University Health Centre Research Ethics Board approved the study. The proband signed an informed consent authorizing genetic analysis in a research setting.

### RNA-sequencing

RNA was extracted from muscle biopsies using TRIzol. The sequencing library was prepared using the TruSeq stranded mRNA library preparation kit and sequenced on an Illumina HiSeq 2500 using 125 bp paired-end reads. Alignment was performed using STAR against a reference genome (hg19) before variant calling with GATK (v.3.7)^21,22^. VCF annotation utilized ANNOVAR databases and custom scripts^23^. Read counts were obtained using featureCounts prior to DESeq differential expression quantification. EnhancedVolcano was used to produce the plot^24^, DEXseq enabled the exon usage analysis^25^, and gene enrichment analysis was performed using GSEA^10–12,26^.

### Reverse transcription polymerase chain reaction

cDNA was obtained following reverse transcription with SuperScript Vilo mix (Invitrogen, 11754050), and amplified with EasyTaq polymerase (TransGen Biotech, AP111–01) using custom primers GGGAATTCGAAGCAAACAAA; GGGGACCTTGAAGGAGAATC. Three nanograms of samples were loaded on a 2% E-gel with 500 ng of 1 Kb Plus E-gel DNA ladder.

### Quantitative polymerase chain reaction

qPCR utilized four different TaqMan probes (Invitrogen): Hs00951675_m1 and Hs00370866_m1 respectively target the exon junctions 5–6 and 9–10 of *MLIP*. Hs00153462_m1 is specific to junctions 2–3 of *LMNA*. Hs03004310_g1 binds junction 1–2 of housekeeping gene *RPS29* for data normalization. Expression levels were assessed through relative ΔΔCT quantification from the QuantStudio 6/7 System.

### Western blot

Proteins were obtained from muscle tissues following TRIzol processing. Thirty micrograms of proteins were loaded on a gradient 4–15% polyacrylamide gel (Bio-Rad, 4561084). Samples were transferred on a PVDF membrane and blocked in 5% milk-PBS (30 min). Antibodies used in this report: MLIP rabbit-polyclonal (Invitrogen PA5–72759, 1:2000 dilution); LMNA rabbit-polyclonal (NEB 2026 S, 1:1000 dilution); GAPDH rabbit-monoclonal (NEB 5174 S, 1:1000 dilution); HRP anti-rabbit (Jackson ImmunoResearch 111–035–114, 1:10000 dilution). Primary antibodies incubation was done overnight at 4 °C with dilutions in 1% BSA-0.1% sodium azide-PBS, followed by 1 h incubation with secondary antibody solution. Proteins revelation with an ECL substrate kit was followed by chemiluminescence capture through the ChemiDoc System, and images were analyzed with the Image Lab 6.0.1 software.

### Long-read sequencing

The library was prepared from total muscle RNA, reverse transcribed with the SuperScript Vilo mix, and amplified (30 cycles) using the LongAmp *Taq* DNA Polymerase (NEB, M0323S). Two sets of barcoded primers targeted exons 3 (TGGACTCCGAAGGGGAAGAT) and 11 (GGGCGAATTACTCCAGGCTT). The amplicons were incubated with the Exonuclease I (NEB, M0293S) for single-stranded DNA cleanup. The library was end prepped with the NEBNext Ultra II End Repair/dA-Tailing Module to allow AMX adapter ligation with the NEBNext Quick Ligation Module ligase (ONT, SQK-LSK109). Purification with AMPure XP beads preceded the final 1× dsDNA QuBit4 fluorometric quantification, which allowed concentration-specific multiplexing of samples. The library was loaded on a flow cell (FLO-MIN106) for MinION sequencing over a 24 h period. The final data were processed with Guppy high-accuracy base calling and demultiplexing, followed by the FLAIR pipeline^27,28^.

### Reporting summary

Further information on research design is available in the Nature Research Reporting Summary linked to this article.

## Supplementary information

Supplementary materials

Reporting Summary

## Data Availability

Data that support the findings of this study are available from the corresponding author upon request. The sequencing data of this study were stored in NCBI Bio Project (PRJNA809363).
