# Supplementary material for: Novel homozygous nonsense mutation of MLIP and compensatory alternative splicing
Source: NPJ Genom Med. 2022 Jun 7;7:36. doi: 10.1038/s41525-022-00307-y (PMC9174206; doi:10.1038/s41525-022-00307-y)
Supplement: Supplementary file 1 — Supplementary materials [file 41525_2022_307_MOESM1_ESM.pdf]

Supplementary materials for

Novel homozygous nonsense mutation of MLIP and compensatory alternative splicing

Jean Mezreani,[1,2†] Sébastien Audet,[1,2†] Florence Martin,[1,2] Jade Charbonneau,[1] Valérie Triassi,[1,3] Eric Bareke,[1] Annie Laplante,[1] Jason Karamchandani,[4,5] Rami Massie,[4,5] Colin H Chalk,[4,5] Erin O'Ferrall,[4,5] and Martine Tétreault [1,2,3]

[†] These authors contributed equally

1 CHUM Research Center, Montreal, Quebec, Canada

2 Department of Neurosciences, University of Montreal, Montreal, Quebec, Canada

3 Department of Bioinformatics, University of Montreal, Montreal, Quebec, Canada

4 Department of Neurology and Neurosurgery, Montreal Neurological Institute, Montreal, Quebec, Canada

5 Department of Pathology, Montreal Neurological Institute, Montreal, Quebec, Canada

Correspondence to: Martine Tétreault

Healthcare Center of the University of Montreal, Research Center Building

CRCHUM, 900 Saint-Denis

Montréal H2X 0A9, Quebec, Canada

E-mail: martine.tetreault@umontreal.ca

Supplementary Table 1. Genetic analysis performed for patient Z46.

Table 1. Clinical Genetic Testing

Gene	Mutation	Zygosity	Status	Associated Disease(s)	Inheritance(s)	Matching Phenotype	Source
RAPSN	c.264C > A	Heterozygous	Pathogenic / Likely Pathogenic	Congenital Myasthenic Syndrome 11 (CMS11)	Autosomal Recessive	No	Gene Panel[1] & RNA-seq
SCN4A	c.4270 G > A	Heterozygous	Pathogenic / Likely Pathogenic	Hyperkalemic Periodic Paralysis (HYPP) - Paramyotonia Congenita (PMC)	Autosomal Dominant - Autosomal Dominant	No	Gene Panel[1] & RNA-seq
MLIP	c.2284 C > T	Homozygous	VUS	–	–	–	RNA-seq
TTN	c.38002 A > G	Heterozygous	VUS	Dilated Cardiomyopathy 1G (CMD1G) - Limb-Girdle, type 2J (LGMD2J)	Autosomal Dominant - Autosomal Recessive	No	RNA-seq
GMPPB	c.893 G > A	Heterozygous	Likely Pathogenic	Muscular Dystrophy-Dystroglycanopathy Type A14 - C14 (MDDGA14 - MDDGC14)	Autosomal Recessive - Autosomal Recessive	No	RNA-seq
LMOD3	c.39_41Del	Heterozygous	Benign	Nemaline Myopathy 10 (NEM10)	Autosomal Recessive	No	RNA-seq
AMPD1	c.303-1 G > A	Heterozygous	VUS	Myopathy Due to Myoadenylate Deaminase Deficiency (MMDD)	Autosomal Recessive	Moderate	RNA-seq
KIF21A	c.3581 C > G	Heterozygous	Benign / Likely Benign	Congenital Fibrosis of the Extraocular Muscles (FEOM)	Autosomal Dominant / Recessive	No	RNA-seq

Negative results from gene panel and RNA-seq analysis: AARS2; ACADS; ACADVL; ACTA1; ADCK3; AFG3L2; AGK; AGL; AGRN; AIFM1; ALDOA; ANO5; ATP2A1; B3GALNT2; B3GNT1; BAG3; BIN1; C10ORF2; C12ORF65; CACNA1S; CAPN3; CAV3; CCDC78; CFL2; CHAT; CHCHD10; CHKB; CHRNA1; CHRNB1; CHRND; CHRNE; CLCN1; CNBP; CNTN1; COA5; COL4A1; COL6A1; COL6A2; COL6A3; COLQ; COQ2; COQ6; COQ9; COX10; COX14; COX6B1; CPT2; CRYAB; DES; DMD; DMPK; DNA2; DNAJB6; DNM2; DOK7; DUX4; DYSF; EARS2; EMD; ENO3; FARS2; FASTKD2; FHL1; FKRP; FKTN; FLNC; FOXRED1; GAA; GBE1; GFER; GFM1; GFPT1; GNE; GTDC2; GYG1; GYS1; HADHA; HADHB; HCCS; HINT1; HNRNPA1; HNRNPA2B1; HRAS; HSPG2; ISCU; ISPD; ITGA7; KABTBD13; LAMA2; LAMP2; LARGE; LDB3; LMNA; LPIN1; MATR3; MEGF10; MGME1; MPV17; MRPL3; MRPS16; MRPS22; MTFMT; MTM1; MTMR14; MTO1; MUSK; MYF6; MYH14; MYH2; MYH7; MYH7B; MYL2; MYOT; NEB; OPA1; PABPN1; PDSS1; PDSS2; PFKM; PGAM2; PGK1; PHKA1; PLEC; PNPLA2; PNPT1; POLG; POLG2; POMGNT1; POMT1; POMT2; PRKAG2; PRKAG3; PTRF; PUS1; PYGM; RBCK1; RMND1; RRM2B; RYR1; SARS2; SCO1; SCO2; SDHAF1; SEPN1; SEPT9; SGCA; SGCB; SGCD; SGCG; SGK196; SIL1; SLC22A5; SLC25A4; SLC37A4; SLC52A1; SLC52A2; SMCHD1; STIM1; SUSLG1; SURF1; SYNE1; SYNE2; TAZ; TCAP; TK2; TNNT1; TNPO3; TPM2; TPM3; TRIM32; TRMU; TSFM; TUBB3; TUFM; TYMP; VCP; YARS2

Additional negative results (RNA-seq analysis only): BYN1, CLC1; COQ10; CYBA; CYBB; DAG1; DMM2; DNPK; ETF; ETFH; HNRPDL; KBTBD13; KLHL4; KLHL40; KLHL41; LBD3; LIMS2; MYPN; OCTN2; POGLUT1; POPDC1; SPEC; TIA1; TOMP1; TOR1AIP1; TRAPPC11; ZNF9

[1] Panel from Medical Neurogenetics LLC. Intron/exon junction coverage ≥ 99.0

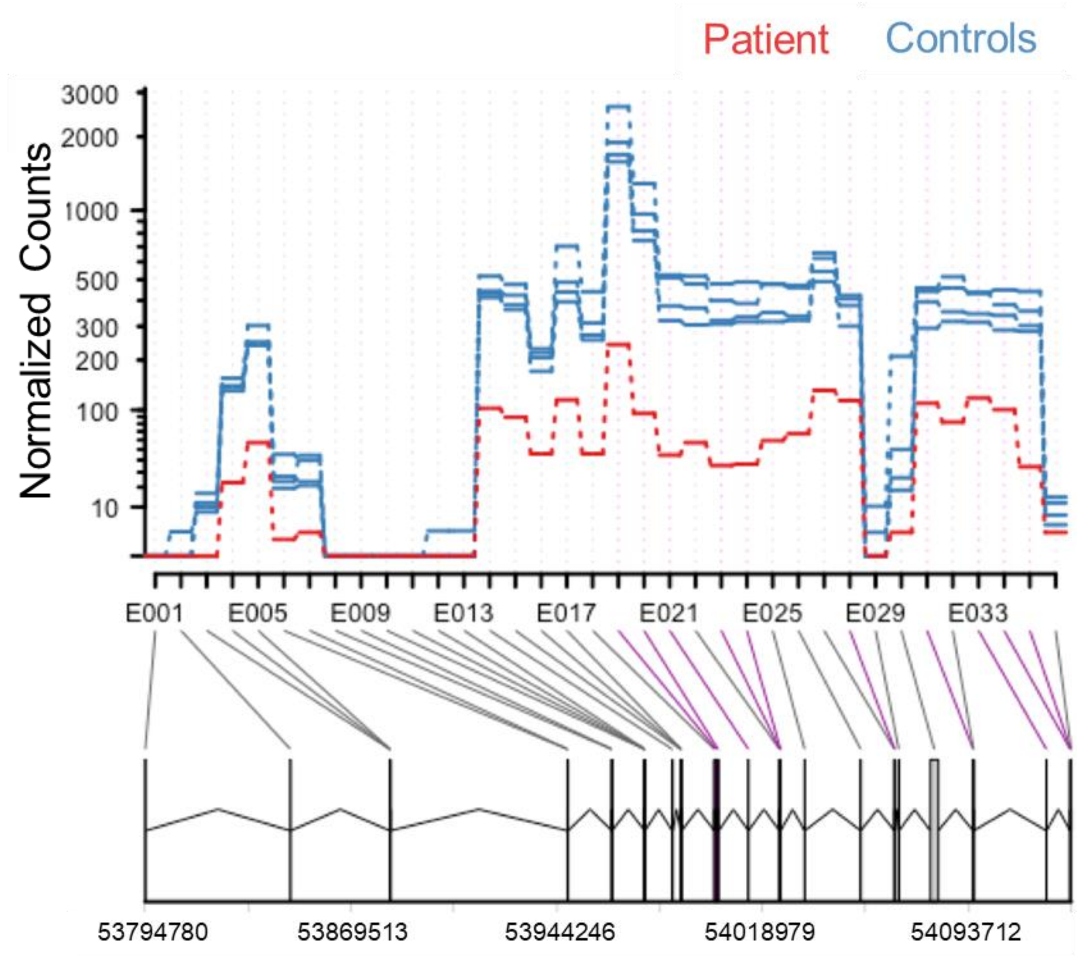

Supplementary Figure 2. Exon usage of MLIP. Exon usage analysis was conducted using DEXSeq, where Ensembl transcripts were collapsed. The flattening approach generates a singular transcript with 36 exons, where exon 5 of MLIP corresponds to either exon 18, 19 or 20 in the representation. When comparing the affected individuals with the healthy samples, a general downregulation of *MLIP* levels can be noted (≈ 3-5 fold), and is slightly emphasized around exon 5 (≈ 8-10 fold).

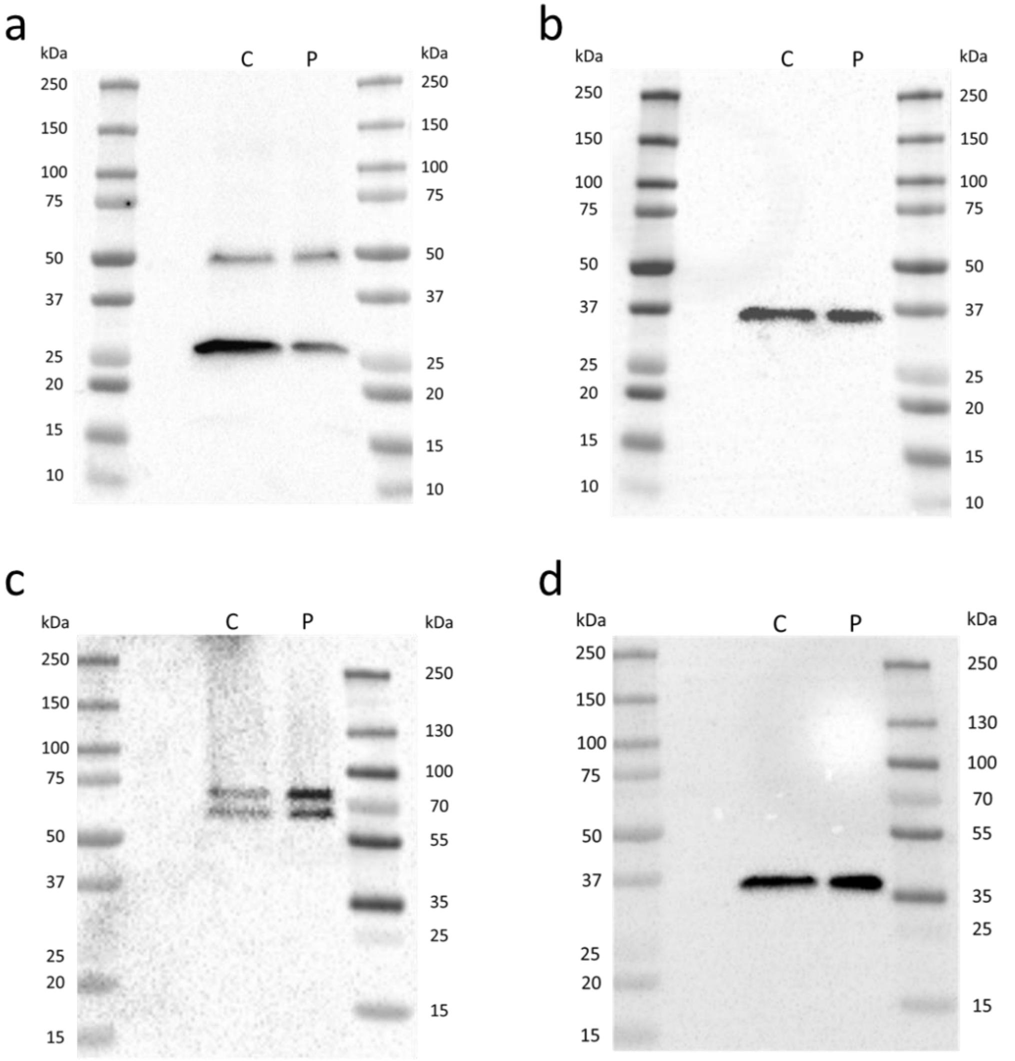

Supplementary Figure 3. *MLIP* and *LMNA* respective expression in muscular tissue (**A**) Western Blot analysis reveals the presence of four isoforms of *MLIP*, two of which can be quantified (27kDa and 50kDa). (**C)** Western Blot analysis reveals the presence of the two isoforms of *LMNA*, lamin A (75kDa) and lamin C (65kDa) were analyzed separately. **(B, D**) *GAPDH* is used as the reference gene for normalization. Full unaltered membranes are shown. Precision Plus Protein Dual Color Standards was used for all blots. PageRuler Plus Prestained Protein Ladder was added for the second blot (**C, D**: Right ladder).
