# Supplementary material for: Novel homozygous nonsense mutation of MLIP and compensatory alternative splicing
Source: NPJ Genom Med. 2022 Jun 7;7:36. doi: 10.1038/s41525-022-00307-y (PMC9174206; doi:10.1038/s41525-022-00307-y)
Supplement: Supplementary file 2 — Reporting Summary [file 41525_2022_307_MOESM2_ESM.pdf]

Corresponding author(s): Martine Tétreault

Last updated by author(s): Apr 11, 2022

Reporting Summary

Nature Portfolio wishes to improve the reproducibility of the work that we publish. This form provides structure for consistency and transparency in reporting. For further information on Nature Portfolio policies, see our Editorial Policies and the Editorial Policy Checklist.

Statistics

For all statistical analyses, confirm that the following items are present in the figure legend, table legend, main text, or Methods section.

n/a	Confirmed	
☒	☐	The exact sample size (*n*) for each experimental group/condition, given as a discrete number and unit of measurement
☐	☒	A statement on whether measurements were taken from distinct samples or whether the same sample was measured repeatedly
☒	☐	The statistical test(s) used AND whether they are one- or two-sided<br>*Only common tests should be described solely by name; describe more complex techniques in the Methods section.*
☒	☐	A description of all covariates tested
☒	☐	A description of any assumptions or corrections, such as tests of normality and adjustment for multiple comparisons
☒	☐	A full description of the statistical parameters including central tendency (e.g. means) or other basic estimates (e.g. regression coefficient) AND variation (e.g. standard deviation) or associated estimates of uncertainty (e.g. confidence intervals)
☒	☐	For null hypothesis testing, the test statistic (e.g. *F*, *t*, *r*) with confidence intervals, effect sizes, degrees of freedom and *P* value noted<br>*Give P values as exact values whenever suitable.*
☒	☐	For Bayesian analysis, information on the choice of priors and Markov chain Monte Carlo settings
☒	☐	For hierarchical and complex designs, identification of the appropriate level for tests and full reporting of outcomes
☒	☐	Estimates of effect sizes (e.g. Cohen's *d*, Pearson's *r*), indicating how they were calculated

Our web collection on statistics for biologists contains articles on many of the points above.

Software and code

Policy information about availability of computer code

Data collection	qPCR data collected with QuantStudio 6 and 7 Flex Real-Time PCR System Software. Western Blot data collected with Bio-Rad Chemi-Doc & Image-Lab Software. LRS data collected using Oxford Nanopore MinKNOW software.
Data analysis	RNA-seq data were analyzed following the GATK Best Practices recommendations. Blots were analyzed using the Image-Lab Software. Tools used for data analysis, such as FLAIR for LRS data, are mentioned in the method section of the paper. Custom codes are a negligible part of the work.

For manuscripts utilizing custom algorithms or software that are central to the research but not yet described in published literature, software must be made available to editors and reviewers. We strongly encourage code deposition in a community repository (e.g. GitHub). See the Nature Portfolio guidelines for submitting code & software for further information.

Data

Policy information about availability of data

All manuscripts must include a data availability statement. This statement should provide the following information, where applicable:

- Accession codes, unique identifiers, or web links for publicly available datasets
- A description of any restrictions on data availability
- For clinical datasets or third party data, please ensure that the statement adheres to our policy

The sequencing data of this study were stored in NCBI Bio Project (PRJNA809363).

Field-specific reporting

Please select the one below that is the best fit for your research. If you are not sure, read the appropriate sections before making your selection.

☒ Life sciences ☐ Behavioural & social sciences ☐ Ecological, evolutionary & environmental sciences

For a reference copy of the document with all sections, see nature.com/documents/nr-reporting-summary-flat.pdf

Life sciences study design

All studies must disclose on these points even when the disclosure is negative.

Sample size	Single patient study with matching healthy control.
Data exclusions	N/A
Replication	qPCR & WB replicated. LRS not replicated due to great amount of data generated and high cost of replication.
Randomization	N/A
Blinding	N/A

Reporting for specific materials, systems and methods

We require information from authors about some types of materials, experimental systems and methods used in many studies. Here, indicate whether each material, system or method listed is relevant to your study. If you are not sure if a list item applies to your research, read the appropriate section before selecting a response.

Materials & experimental systems

n/a	Involved in the study	
☐	☒	Antibodies
☒	☐	Eukaryotic cell lines
☒	☐	Palaeontology and archaeology
☒	☐	Animals and other organisms
☐	☒	Human research participants
☒	☐	Clinical data
☒	☐	Dual use research of concern

Methods

n/a	Involved in the study	
☒	☐	ChIP-seq
☒	☐	Flow cytometry
☒	☐	MRI-based neuroimaging

Antibodies

Antibodies used	MLIP (Invitrogen, PA5-72759); LMNA (NEB, 2026S); GAPDH (NEB, 5174S); Anti-Rabbit (Jackson ImmunoResearch, 111-035-114)
Validation	All primary antibodies are validated for use against human proteins. All antibodies, except for MLIP, are validated for use with Western blots. MLIP antibody is validated for immunohistochemistry.

Human research participants

Policy information about studies involving human research participants

Population characteristics	Single patient with late-onset myopathy without a genetic cause.
Recruitment	Recruited for RNA-seq following failed attempt to identify genetic cause with gene panels.
Ethics oversight	McGill University Health Centre Research Ethics Board

Note that full information on the approval of the study protocol must also be provided in the manuscript.
